# Corneal Power, Anterior Segment Length and Lens Power in 14-year-old Chinese Children: the Anyang Childhood Eye Study

**DOI:** 10.1038/srep20243

**Published:** 2016-02-01

**Authors:** Shi-Ming Li, Rafael Iribarren, Meng-Tian Kang, He Li, Si-Yuan Li, Luo-Ru Liu, Yun-Yun Sun, Bo Meng, Si-Yan Zhan, Jos J. Rozema, Ningli Wang

**Affiliations:** 1 Beijing Tongren Eye Center, Beijing Tongren Hospital, Capital Medical University, Beijing, China; 2 Department of Ophthalmology, San Luis Medical Center, Buenos Aires, Argentina; 3 Anyang Eye Hospital, Henan Province, China; 4 Department of Epidemiology and Health Statistics, Peking University School of Public Health, Beijing, China; 5 Department of Ophthalmology, Antwerp University Hospital, Edegem, Belgium; 6 Department of Medicine and Health Sciences, Antwerp University, Wilrijk, Belgium

## Abstract

To analyze the components of young Chinese eyes with special attention to differences in corneal power, anterior segment length and lens power. Cycloplegic refractions and ocular biometry with LENSTAR were used to calculate lens power with Bennett’s method. Mean refraction and mean values for the ocular components of five different refractive groups were studied with ANOVA and post-hoc Scheffé tests. There were 1889 subjects included with full data of refraction and ocular components. As expected, mean axial length was significantly longer in myopic eyes compared to emmetropes. Girls had steeper corneas, more powerful lenses and shorter eyes than boys. Lens power was lower in boys and also lower in myopic eyes. Lens thickness was the same for both genders but was lower in myopic eyes. Although cornea was steeper in myopic eyes in the whole sample, this was a gender effect (more girls in the myopic group) as this difference disappeared when the analysis was split by gender. Anterior segment length was longer in myopic eyes. In conclusion, myopic eyes have lower lens power and longer anterior segment length, that partially compensate their longer axial length. When analyzed by gender, the corneal power is not greater in low and moderate myopic eyes.

During the first years of ocular growth in humans, the cornea and the lens lose power while the axial length grows, increasing the anterior segment and the vitreous chamber lengths. During this period refraction remains stable and becomes clustered in a leptokurtic low hyperopic distribution because more hyperopic eyes tend to grow faster in axial length^1^. This emmetropization process is based on the fact that hyperopic defocus is a potent stimulator of the rate of ocular growth^2^.

After age 4–6, at school entrance, myopic cases appear in the clinic. This annual incidence of myopia can be low or high according to different environments. In rural settings it is usually low (1–3% incidence per year) but can be as high as 10% per year cumulative incidence in a city like Anyang, in mainland China, where this study was conducted^3^. By age 14, more than seventy percent of the children are myopic in this city^3^, making this population interesting for studying myopia development.

Previous prospective studies on the changes of the ocular components during myopia development^4^,^5^,^6^,^7^,^8^,^9^ have shown that the principal change seen is an increased rate of axial elongation in myopic cases. For this reason most myopia studies have considered changes in axial elongation, and paid substantially less attention to the anterior segment (cornea, lens and anterior segment length).

The corneal power remains the same for all ages after age 3 in many cross-sectional population based studies, and during school ages and myopia development in these prospective studies mentioned^4^,^5^,^6^,^7^,^8^. The lens loses power rapidly before age 10 and then more slowly through most of life^10^. The cornea has been shown to be steeper in myopic eyes since the time of Steiger^11^. He had found that the corneal power had a normal distribution and his studies were the basis for later studies of the ocular components. He showed that myopic eyes had one diopter more corneal power than emmetropic eyes^11^,^12^. Sorsby *et al.* found similar data in his classical cross-sectional study, concluding that a steeper cornea in myopic eyes could contribute to myopia development^13^. Scott and Grosvenor^14^ also found steeper corneal power in myopic eyes when they developed a model for ocular growth based on axial length/corneal radius ratio.

Besides, longitudinal studies have shown lens thinning in children, which is often accompanied by anterior chamber deepening^1^,^4^,^6^,^15^. Also cross-sectional studies have shown thinner lenses in myopic eyes^9^,^16^. But the relation between the anterior segment length growth and myopia development has not been carefully studied. Since most of the data in the literature focused on changes at the posterior pole, instead this paper looks at the anterior ocular components in myopic eyes, with special attention to differences in corneal power, anterior segment length and lens power between different refractive groups. Given the known differences in these parameters between genders (women have steeper corneas, shorter axial lengths, more powerful lenses, and smaller anterior segment lengths than men)^17^, separate analyses are presented for the entire sample and both genders.

## Results

Of the 2267 subjects examined, a total of 1889 (83.3%) subjects had all data required for this study. Of these, girls accounted for 51.3%. Their mean age and ocular components are given in Table 1, where it can be seen that girls had shorter eyes, with greater corneal and lens powers.

**Figure 1:**
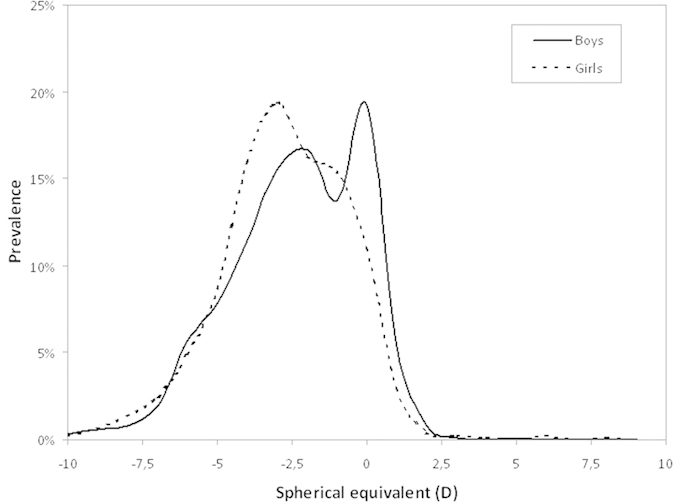
Prevalence of refractive error split by gender. It can be seen the girls are more frequent in the low and moderate myopia group while boys are more frequent in the emmetropic category.

The number of subjects in each refractive category are presented in Table 2 and Fig. 1, where it can be seen that girls were more frequent in the myopic group, while boys were more prevalent in the emmetropic and hyperopic categories. Table 3 shows the mean values of the refractive error and ocular components of the different refractive groups, which indicates that the main difference among refractive groups relied in the axial length. Moreover there were significant differences in mean corneal power between myopes and emmetropes, having myopic subjects higher corneal power (p < 0.001). The lens power was lower in myopes and higher in hyperopes when compared to emmetropes (p < 0.001). The lens thickness and the anterior chamber depth showed known differences^18^ between refractive groups (p < 0.001), but the anterior segment length was significantly greater only in myopic subjects (p < 0.001). The white to white corneal diameter was similar for the different refractive groups.

Table 4 shows the mean ocular components for the group of 327 emmetropic children. It can be seen that boys have longer eyes, with lower corneal and lens powers, with deeper anterior segment lengths, deeper anterior chamber depths and larger white to white corneal diameters (all p < 0.001), but similar lens thickness as girls. Since such differences in mean ocular components between genders could influence the refractive groups outcomes in Table 3, the following analysis was split by gender.

**Figure 2:**
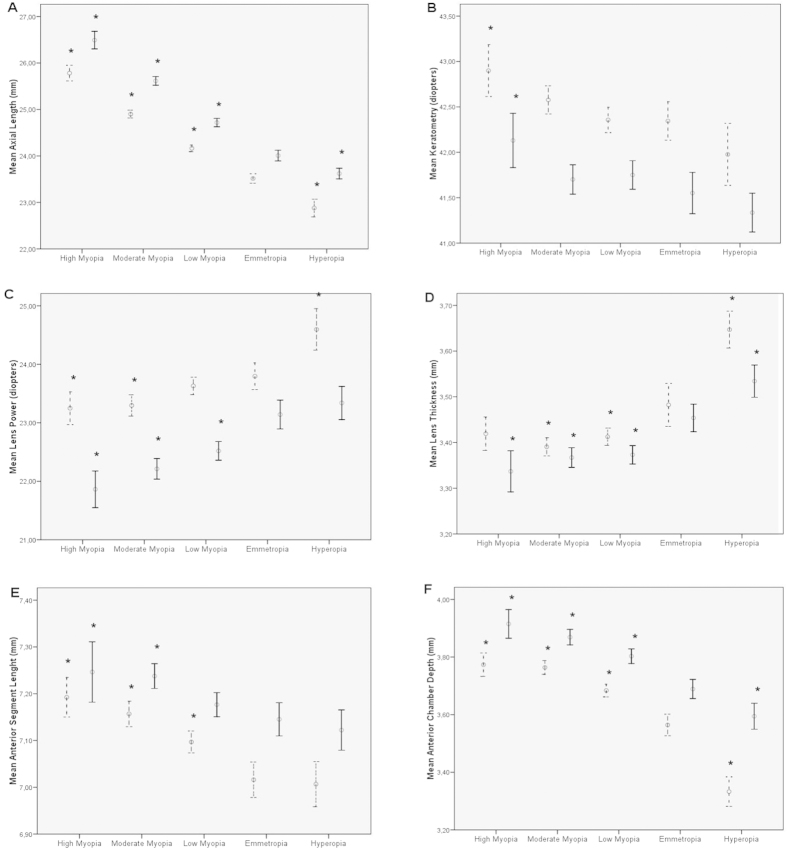
Mean ocular components and 95% confidence intervals for the different refractive error groups. Girls in doted lines, boys in full lines. The asteriscs (*) represent significant differences (p < 0.001) when the given group is compared to the emmetropic group of the same gender. The whiskers with the 95% confidence intervals also show the significance of the differences when they do not reach the mean of the group with which any given mean is compared.

Figure 2 shows the ocular components for girls and boys. As expected, both myopic girls and boys had significantly longer axial lengths than their emmetropic peers (p < 0.001 both cases, Fig. 2A). But myopic girls or myopic boys as a whole (i.e. SE < 0.5D) did not differ in keratometry (p = 0.374 and p = 0.135, respectively) compared to their emmetropic peers (Fig. 2B). The only significant differences in keratometry for this analysis split by gender was found between myopes as a whole (SE < 0.5D) and hyperopes (p = 0.009 for girls and p = 0.004 for boys) and for highly myopic girls or boys when compared to emmetropes (p = 0.049 and 0.035 respectively, Fig. 2B).

Both in boys and girls the lens power was lower in myopic subjects (SE < 0.5D) when compared to emmetropes (p < 0.001 in both cases, Fig. 2C). When split by gender this difference in lens power respect emmetropic peers was less pronounced for myopic girls than for boys (Fig. 2C). For example, the difference in lens power between emmetropes and moderate myopic girls was –0.50 D and for moderate myopic boys was –0.93 D (Fig. 2C). The lens power was higher only in hyperopic girls (p < 0.001) and not in hyperopic boys (p = 0.558) when compared to their emmetropic peers (Fig. 2C). In both genders the lens was thinner in myopic eyes and thicker in hyperopic eyes (Fig. 2D). Interestingly, the anterior segment length was similar for emmetropic and hyperopic eyes, but bigger in all myopic eyes (boys and girls, p < 0.001 both cases, Fig. 2E). The white to white corneal diameter was greater in boys (Table 4) but there were no differences in this parameter between refractive groups, both for boys and for girls (data not shown). Figure 2 F shows the greater anterior chamber depth in boys and myopic groups.

## Discussion

This study analyzed a representative sample of children from mainland China with high prevalence of myopia by age 14. The myopic category included more girls than boys. Boys, on the other hand, were more present in the emmetropic or hyperopic categories. Already since the time when keratometry was developed in the 19^th^ century, myopic subjects were found to have steeper corneal powers than their emmetropic peers^11^,^12^. Moreover, myopes have consistently been shown to have lower lens powers in previous studies on lens power calculation. As girls have shorter eyes, with more curved lenses and steeper corneas, the difference in prevalence of genders in the different refractive groups could influence the analysis of the ocular components by refractive groups. This was seen in the analysis split by gender, which showed that the steeper corneas found in myopic subjects was in part a gender effect. Here we found, for the first time to our knowledge, that there were small non-significant differences in corneal power between low or moderate myopes vs. emmetropes, both for boys and girls when treated separately.

The reason as to why high myopes still have significantly steeper corneas than emmetropes or hyperopes (even when split by gender) remains unclear. Shorter emmetropic eyes tend to have steeper corneas, and if they were more prone to develop myopia when the rate of axial elongation is increased due to environmental reasons, this would result in myopic eyes having higher corneal powers. Meanwhile hyperopic children born with short eyes and flat corneas may not have emmetropized correctly and remained hyperopic, thus increasing the observed difference in corneal power between myopes and hyperopes.

An alternative explanation for the steeper corneas in myopes was given by Scott and Grosvenor^19^, who, following van-Alphen^20^, proposed that during myopia development the axial stretching could cause the cornea to become steeper. However this model does not agree with recent observations in prospective, longitudinal studies following newly developed and progressive myopes, where the cornea is actually seen to flatten slightly with increasing myopia^21^. Also, recent prospective data from the longest longitudinal study of myopic children shows that the cornea flattens slightly with myopia development^22^. The explanation by Scott and Grosvenor is therefore unlikely to be correct.

Concerning the lens, Mutti *et al.*^18^. showed that girls had lenses with steeper curvatures and higher lens power than boys. The present study also showed that girls, in the emmetropic category, had more powerful lenses than boys, but with similar lens thickness. In both genders the lens was thinner and less powerful in myopic eyes; but there was a –0.35D difference in lens power for myopic girls as a whole (p = 0.03) and a –0.83D difference for myopic boys as a whole, when compared to emmetropes (p < 0.001). These cross-sectional data show that perhaps the lens loses more power in boys than in girls during myopia development. Interestingly, the Shahroud Study has shown differences between genders in the change in lens power with age in adults: with cross-sectional data, hyperopic women showed greater decreases in lens power with ageing when compared to hyperopic men^17^. In the present study there were also differences in the lens power between genders in the hyperopic category: while hyperopic girls had significantly higher lens powers by +0.80D (p = 0.002), the hyperopic boys only had +0.20D higher lens power than their emmetropic peers (non-significant difference, p = 0.541).

The causes of these differences are not clear, and more research is needed to explain these findings in lens power between genders. It is interesting to note that the difference in lens power between boys and girls is not related to lens thickness, but that the difference in lens power between myopes and emmetropes is related to lower lens thickness in myopic eyes. It has been argued that decreased lens growth during myopia development alters the gradient index structure of the lens^10^. The present data show that the more curved lenses in smaller eyes of girls are less prone to have lower power in myopic eyes, adding another variable to this problem and perhaps explaining why myopia is more pronounced in girls. Future longitudinal data from the present study may show how these differences in lens power loss among genders develop with the onset of myopia.

The anterior segment length differences are also interesting. From magnetic resonance imaging^23^ and biometry^24^ it is clear that the main ocular component involved in ocular growth during myopia development is the vitreous chamber depth. The corneal power has been shown to be stable during ocular growth after age 3, so it would not change much during myopia development if the main changes are found in the vitreous chamber. But bigger anterior segment lengths in the myopic subjects of this study raise the possibility that the anterior segment is involved to some extent in ocular growth during myopia development. The anterior segment length depends on the anterior chamber depth, the lens thickness, and the position of the lens in its place by the ciliary muscle and the zonulae. Scleral growth in the zone between the limbus and the insertion of the ciliary muscle could increase the anterior segment length in myopic children. Alternatively, subtle differences in the ciliary muscle thickness have been shown in myopes when compared to emmetropes^25^. Myopes have thicker posterior ciliary muscles than emmetropes^26^,^27^. It is then possible that thicker posterior ciliary muscles exert some tension on the posterior zonular fibers that puts the lens in a backward position in the anterior segment, thus increasing the anterior segment length without changes in the equatorial scleral growth. In support of this idea, a prospective study in schoolchildren showed that during ocular growth at myopia development, the anterior segment cord (located in the posterior chamber) did not increase in diameter with age^28^.

In this study, the actual amount of increased anterior segment length in myopic eyes was small, in the order of +0.12 mm for girls and +0.07 mm for boys (Fig. 2E). The scleral sector involving possible ocular growth at the insertion of the ciliary muscle extends from the scleral spur to the base of the ciliary muscle, approximately 1.5 mm^29^. Then, the change in anterior segment length of myopic eyes would involve a growth of about 7% of this scleral sector where the ciliary muscle is attached. When myopia develops the axial length extends beyond normal range by 1–2 mm for low and moderate cases of myopia, mainly by growth of the vitreous chamber. The amount of compensation of axial myopia by this backward placement of the lens is also small, in the order of 0.3 diopters at three diopters per mm change in axial length. In the case of girls, for example the difference of +1.17 mm in axial length of myopic vs. emmetropic eyes would represent a change in refraction of –3.51 diopters, while the actual difference in refraction of myopic girls was –3.06 diopters. This lower than expected myopia development was compensated by the lower lens power and the backward position of the lens.

In conclusion, myopic eyes have lower lens power and longer anterior segments lengths, that compensate in part their longer axial length. Although previous reports had shown steeper corneal curvature in myopic eyes, in this study the corneal power was not greater in low and moderate myopic eyes when the analysis was split by gender. It is not clear yet whether the anterior segment growth could be related to the vitreous chamber elongation of myopic eyes. Future studies about the ocular components of refraction should be split by gender.

## Methods

### Study Population

The Anyang Childhood Eye Study (ACES) is a cohort study aimed to observe the prevalence, incidence and risk factors for myopia among Chinese children in urban areas of Anyang city, Henan Province, Central China. The ACES was approved by the Ethics Committee of Beijing Tongren Hospital, Capital Medical University, and adhered to the tenets of the Declaration of Helsinki. Each child was asked for verbal assent, as well as informed written consent from at least one parent. Details of the survey methods have been given elsewhere^3^. In brief, 2267 grade 7 students from 4 junior middle schools were randomly selected using stratified cluster sampling and were examined from October to December 2011.

### Eye Examinations

Cycloplegic autorefraction was performed 30 minutes after 2 drops of 1% cyclopentolate (Alcon) and 1 drop of 0.5% tropicamide (Mydrin P, Santen, Japan). The average of three measurements automatically performed by the instrument (HRK-7000A, Huvitz, Korea) was used for analyses. Refraction was defined as spherical equivalent (*SE*, sphere power + cylinder power/2). Myopia was defined as *SE* < –0.5D, hyperopia as *SE* > +0.5D and emmetropia as –0.5 ≤ *SE* ≤ +0.5D. Besides, low myopia was defined as that between SE < –0.50 D and –2.50 D, moderate myopia between <–2.50 D and –5.00 D and high myopia that <–5.00 D.

Biometric parameters, including axial length, anterior chamber depth, lens thickness, central corneal thickness, corneal diameter and (anterior) corneal radius of curvature, were measured using Lenstar LS900 (Haag-Streit Koeniz, Switzerland). Five repeated measurements were taken and averaged. Mean corneal radius of curvature was calculated as the average of the greatest and the least corneal radius of curvature. Corneal power was calculated from the mean anterior corneal radius of curvature with 1.328 ideal index^30^,^31^,^32^. Cycloplegic refractions and the ocular biometry were used to calculate crystalline lens power with Bennett’s formula^33^,^34^. Effective anterior chamber depth for this formula included corneal thickness and LENSTAR given anterior chamber depth. The axial length was corrected to include 0.2 mm of retinal thickness, which is subtracted automatically by the LENSTAR to match the A-Scan biometry used in the intraocular lens calculation regression formulas^16^. Adding retinal thickness redefines axial length as the distance between corneal vertex and the retinal pigment epithelium, which makes the *in-vivo* crystalline lens power calculation more accurate as the optical axial length should be up to the photoreceptor plane rather than the internal limiting membrane, which is the limit for the A-Scan. Anterior segment length was defined as the sum of effective anterior chamber depth and lens thickness.

### Statistical Analyses

Statistical analysis was performed using SPSS software. Given that there were high Pearson correlations between right and left eyes for biometry data and refraction (ranging between 0.87 and 0.96), only the data of right eyes are used for analysis. The refractive groups were used for analysis of variance (ANOVA) with post-hoc Scheffé tests to look at the differences in mean values of corneal power, lens power, axial length, lens thickness, anterior chamber depth and anterior segment length. ANOVA by refractive groups was performed both for the entire sample, and split by gender. P values were considered significant at the 0.05 level.

## Additional Information

**How to cite this article**: Li, S.-M. *et al.* Corneal Power, Anterior Segment Length and Lens Power in 14-year-old Chinese Children: the Anyang Childhood Eye Study. *Sci. Rep.* **6**, 20243; doi: 10.1038/srep20243 (2016).

## Figures and Tables

**Table 1:** Mean values (and SD) for boys and girls in the whole sample.

n = 1889	Boys	Girls	All	p*
Mean Age (years)	13.71 (0.47)	13.66 (0.48)	13.68 (0.48)	0.11
Mean Spherical Equivalent (diopters)	−1.91 (2.19)	−2.29 (2.16)	−2.10 (2.19)	0.001
Mean Keratometry (diopters)	41.68 (1.37)	42.45 (1.37)	42.08 (1.42)	0.001
Mean Axial Length (mm)	24.84 (1.15)	24.38 (1.04)	24.60 (1.12)	0.001
Mean Lens Power (diopters)	22.60 (1.48)	23.57 (1.43)	23.08 (1.54)	0.001
Mean Lens Thickness (mm)	3.40 (0.19)	3.43 (0.19)	3.42 (0.19)	0.003
Mean Anterior Segment Length (mm)	7.19 (0.23)	7.11 (0.22)	7.15 (0.23)	0.001

*p value of the difference between boys and girls (student t test).

**Table 2:** Number of subjects (%) in each refractive category.

	Boys	Girls
High Myopia < −5.00 D	85 (9.3%)	94 (9.7%)
Moderate Myopia −5.00 D– < −2.50 D	248 (27.0%)	321 (33.0%)
Low Myopia −2.50 D– < −0.50 D	282 (30.8%)	342 (35.2%)
All Myopia < −0.50 D	615 (67.06%)	757 (77.88%)
Emmetropia	178 (19.41%)	149 (15.33%)
Hyperopia > +0.50 D	124 (13.52%)	66 (6.79%)

All differences significant p < 0.001 (chi square).

**Table 3:** Mean ocular components data for the whole sample (SD).

	Myopia < −0.50 D	Emmetropia	Hyperopia > +0.50 D
Mean Spherical Equivalent (diopters)	−3.06* (1.71)	+0.01 (0.33)	+1.20* (0.99)
Mean Keratometry (diopters)	42.18* (1.40)	41.91 (1.49)	41.56* (1.30)
Mean Axial Length (mm)	24.97* (1.0)	23.78 (0.75)	23.36* (0.77)
Mean Lens Power (diopters)	22.91* (1.51)	23.43 (1.50)	23.76 (1.42)
Mean Lens Thickness (mm)	3.39* (0.17)	3.47 (0.23)	3.57* (0.18)
Mean Anterior Segment Length (mm)	7.17* (0.43)	7.09 (0.41)	7.09 (0.49)
Mean Anterior Chamber Depth (mm)	3.78* (0.22)	3.63 (0.22)	
Mean White to White Distance (mm)	12.02 (0.43)	12.02 (0.41)	12.07 (0.49)

*p < 0.001 when compared to emmetropes.

**Table 4:** Mean values (SD) for the ocular components in emmetropic children.

n = 327	Boys	Girls	p
Mean Keratometry (diopters)	41.55 (1.54)	42.35 (1.31)	*
Mean Axial Length (mm)	24.01 (0.78)	23.51 (0.63)	*
Mean Lens Power (diopters)	23.14 (1.59)	23.79 (1.29)	*
Mean Lens Thickness (mm)	3.45 (0.19)	3.48 (0.27)	
Mean Anterior Chamber Depth (mm)	3.69 (0.22)	3.56 (0.22)	*
Mean Anterior Segment Length (mm)	7.14 (0.239	7.01 (0.22)	*
Mean White to White Distance (mm)	12.08 (0.40)	11.95 (0.42)	*

*p < 0.001 Student t test.
