# Corrigendum: Corneal Power, Anterior Segment Length and Lens Power in 14-year-old Chinese Children: the Anyang Childhood Eye Study

**DOI:** 10.1038/srep26919

**Published:** 2016-06-10

**Authors:** Shi-Ming Li, Rafael Iribarren, Meng-Tian Kang, He Li, Si-Yuan Li, Luo-Ru Liu, Yun-Yun Sun, Bo Meng, Si-Yan Zhan, Jos J. Rozema, Ningli Wang

Scientific Reports 6: Article number: 20243; 10.1038/srep20243 published online: 02012016; updated: 06102016.

In the original version of this Article, there were errors in Affiliation 1 which was incorrectly listed as ‘Beijing Tongren Eye Center, Beijing Tongren Hospital, Capital Medical University, Beijing, China.’ The correct affiliation is listed below:

Beijing Tongren Eye Center, Beijing Tongren Hospital, Beijing Ophthalmology Visual Science Key Lab, Beijing Institute of Ophthalmology, Capital Medical University, Beijing, China.

These errors have now been corrected in the PDF and HTML versions of the Article.

